# The Impact of Repeat HIV Testing on Risky Sexual Behavior: Evidence from a Randomized Controlled Trial in Malawi

**DOI:** 10.4172/2155-6113.1000549

**Published:** 2016-03-02

**Authors:** Adeline Delavande, Zachary Wagner, Neeraj Sood

**Affiliations:** 1 Institute for Social and Economic Research, University of Essex, Colchester, UK; 2 School of Public Health, University of California, Berkeley, 50 University Hall, Berkeley, CA, United States; 3 Schaeffer Center for Health Policy and Economics, University of Southern California, Los Angeles, United States

**Keywords:** HIV/AIDS, HIV testing, Malawi, Behavioral economics, Health economics, Serodiscordant couple

## Abstract

A significant proportion of HIV-positive adults in sub-Saharan Africa are in serodiscordant relationships. Identification of such serodiscordant couples through couple HIV testing and counseling (HTC) is thought to promote safe sexual behavior and reduce the probability of within couple seroconversion. However, it is possible HTC benefits are not sustained over time and therefore repeated HTC may be more effective at preventing seroconversion than one time HTC. We tested this theory in Zomba, Malawi by randomly assigning 170 serodiscordant couples to receive repeated HTC and 167 serodiscordant couples to receive one time HTC upon study enrollment (control group). We used linear probability models and probit model with couple fixed effects to assess the impact of the intervention on risky sexual behavior. At one-year follow-up, we found that couples that received repeated HTC reported significantly more condom use. However, we found no difference in rate of seroconversion between groups, nor did we find differences in subjective expectations about seroconversion or false beliefs about HIV, two expected pathways of behavior change. We conclude that repeated HTC may promote safe sexual behavior, but this result should be interpreted with caution, as it is inconsistent with the result from biological and subjective outcomes.

## Introduction

Malawi has one of the highest HIV prevalence in the world with 10% of the adult population being infected [1]. As in many countries in sub-Saharan Africa, heterosexual intercourse is the predominant mode of HIV transmission in Malawi, accounting for 88% of all new infections [2]. Moreover, several studies have shown that a significant proportion of couples in steady relationships have serodiscordant status, ranging, for instance, from 7% in rural Uganda to 21% in Zambia [3-5]. Therefore, finding ways of reducing HIV transmission within serodiscordant couples is critical for controlling the epidemic.

There has been a recent shift towards couples HIV testing and counseling as means for preventing within couple HIV transmission [2]. Becoming aware of one's status not only allows for effective prevention efforts, such as initiation of antiretroviral treatment (ART) and pre-exposure prophylaxis (PrEP)[6,7], but several studies have shown that HTC among serodiscordant couples has been successful in terms of reducing risky sexual behavior [5,8-10].

Although this prior work is encouraging, reductions in risky behavior may not be sustained over time. A recent report from Malawi found that serodiscordant couples who shared their HIV test results were more likely to report condom use but this change was only sustained over a short period of time [11]. This suggests that a longer duration after a couple's HTC session may be associated with greater expectation that seroconversion occurred, reducing the perceived value of condom use. There is little incentive to use condoms if both partners believe they are positive. Therefore, reducing the duration between negative tests by increasing the frequency of couples HTC may prolong safe sexual behavior, and reduce the probability of seroconversion. Moreover, more frequent HTC increases exposure to the valuable HIV prevention information provided by counselors, which may help reinforce safe behavior.

This study is the first to evaluate the impact of repeated HTC among serodiscordant couples. From July 2011 to January 2013, we conducted a randomized controlled trial in Zomba, Malawi, by which half of the enrolled couples were randomly assigned to received repeated (quarterly) HTC and half were assigned to a control group. We measured how repeat HTC affected seroconversion, risky sexual behavior, and subjective beliefs about HIV status.

## Methods

### Study Population and Enrollment

**Figure 1:**
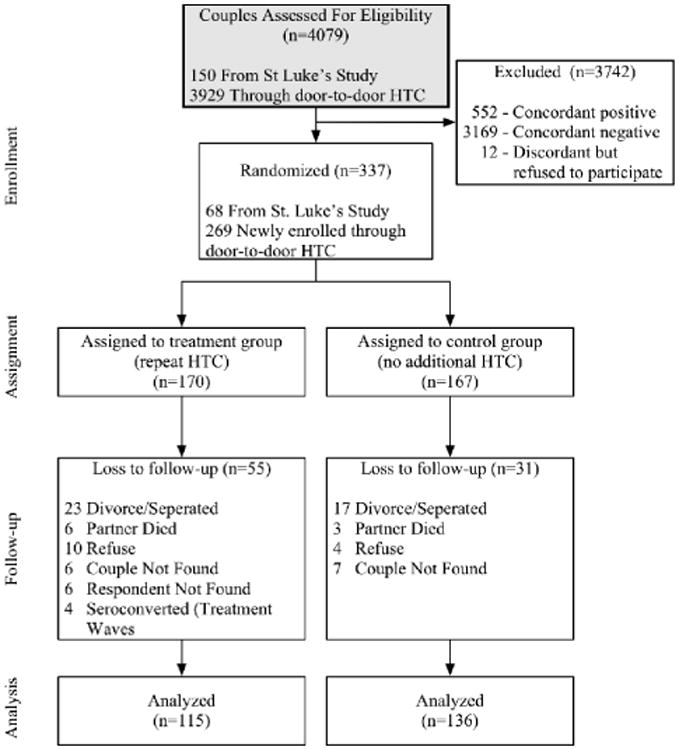
Flow diagram of sample.

The study population was serodiscordant couples living in rural areas of Zomba, Malawi. We enrolled couples to participate in the study in two ways (Figure 1). First, we acquired a list of 3,400 previously identified discordant couples who participated in a prior study in 2011 conducted by St. Luke's Mission Hospital in Zomba, Malawi. In October of 2011, 150 of these serodiscordant couples were re-visited (those with adequate tracking information) and offered another couple HTC session, where both partners were re-tested. Couples that remained serodiscordant (had not divorced or seroconverted) were asked to be enrolled in the study and to complete a baseline survey. Of the 150 couples contacted from the St. Luke's program, 87 were jointly tested and 68 were identified as still serodiscordant. All 68 eligible couples agreed to be enrolled in the study.

Next, we enrolled additional couples through a door-to-door HTC campaign. We trained experienced HIV counselors who travelled door-to-door, providing HTC services to all cohabitating couples with at least one partner between the ages of 15 and 55 across 129 Enumeration Areas (EAs) in Zomba district. EAs were randomly selected from a list of all EAs in Zomba district using a random number generator. Counselors went to all villages within an EA and visited all accessible households within a village. This process resulted in HIV testing of 3,929 couples. Couples found to be serodiscordant were asked to participate in the study and all consenting couples were asked to immediately take part in a baseline survey, which captured information on risky sexual behavior, subjective beliefs about HIV, and other demographic and behavioral information that may affect the probability of risky behavior or HIV transmission. All survey questions were asked to both partners in separate survey sessions. Of the 277 couples that were found to be discordant, 269 agreed to participate in the study for a total of 337 couples (including the St. Luke's group).

### Random Assignment and Intervention

We randomly assigned 170 couples to receive couples HTC every 4 months for the 12-month duration of the study (treatment group) and 167 couples to receive no additional HTC until after study completion (control group) (Figure 1). In other words, couples assigned to the treatment group were visited for HTC 4 months and 8 months after enrollment, whereas couples assigned to the control group were not visited during this period. In order to ensure randomization resulted in balance between treatment and control groups on key characteristics, we employed a re-randomization technique, by which we re-randomized couples until there were no statistical differences across groups in characteristics which we deemed to be important predictors of sexual behavior or seroconversion [12]. We ensured that couples were balanced on the following characteristics: number of children, age, gender of HIV+ member, education, land ownership, monthly income, employment status, intra-couple bargaining power, and risky-sexual behavior.

At each HTC session, both partners were counseled on safe sexual behavior and strategies for effective HIV management, and the HIV negative partner was given an HIV test. HIV tests were conducted with the Determine test kits and positive test results were verified using Uni-Gold and Bioline test kits. Couples in the treatment group who seroconverted during the intervention were dropped from subsequent treatment rounds.

After 12 months, all couples were revisited to complete a follow-up survey similar to the baseline survey and receive a final HTC session (surveys were completed prior to HIV testing). Couples that were no longer cohabiting were not included in the study (although HTC services were still provided). 55 couples assigned to the treatment group and 31 couples assigned to the control group were lost to follow-up. Attrition was more common in the treatment group partly because this group had more visits and thus more opportunities to drop out of the study. In the treatment group, 23 couples divorced, 6 partners died, 10 couples refused to continue with the study, 12 couples were not found, and 4 couples seroconverted during one of the two treatment waves. In the control group, 17 couples divorced, 3 partners died, 4 couples refused, and 7 couples were not found. This left 115 and 136 couples for analysis in the treatment and control group, respectively. We tested for differential attrition by measuring differences in key characteristics among those that attritted between treatment and control groups. We found no evidence that couples that attrited in the treatment group were significantly different from those that attrited in the control group (Appendix Table A1).

### Outcomes and Measurement

We expected repeat testing to reduce seroconversion through two main channels: (i) reducing the perceived probability that seroconversion had occurred, thus creating more perceived benefit from condom use, (ii) being reminded about the effectiveness of condoms at preventing infection during counseling sessions. Therefore we measured the affect of the intervention on the main outcome (seroconversion) as well as the pathways to the main outcome (subjective expectations, knowledge about the disease, and risky sexual behavior).

### Seroconversion

We used HIV test results to measure seroconversion. We classified couples as having seroconverted if the partner who was HIV negative at baseline tested HIV positive at follow-up (or at any of the treatment waves if in the treatment group). We denominated this count data by the total number of people tested in each group in the follow-up HTC session.

### Risky Sexual Behavior

Risky sexual behavior measures were based on a series of questions asking respondents about sexual activity with their spouses. All questions were asked at both baseline and follow-up waves. All outcomes were created at the couple level. If responses were inconsistent within a couple, we used the response that indicated riskier sexual behavior since we expected under-reporting of risky behavior [13]. At follow-up, responses were inconsistent between partners for between 20% and 34% of couples depending on the outcome. There was no difference between treatment and control groups (Appendix Table A2). If the male was HIV positive, both partners were more likely to report safer behavior than if the female was positive. For most measures, the male reported safer measures than the female regardless of who was HIV positive (available upon request).

Counselors first asked respondents if they “usually had sex without a condom” (“yes” or “no”). If either partner reported yes, the couple was classified as usually having had sex without a condom. Next, counselors asked respondents who reported not usually having sex without a condom if they “ever had sex without a condom” (“yes” or “no”). If either partner reported “yes” to this question or the prior question, the couple was classified as having had sex without a condom in the previous months. Next, all respondents were asked how frequently they used a condom in the past few months, with possible responses of never, sometimes, or usually. We classified couples as the least frequent value reported within the couple. Finally, respondents were asked how frequently they had sexual intercourse with their partner; 4+ times per week, 1-3 times per week, a couple of times a month, about once a month, or less than once a month. We classified a couple based on the most frequent value reported within the couple. We expected couples that received repeat HTC to report less risky sexual behavior measures.

### Subjective Expectations and False Beliefs

We expected that repeat testing would reduce couples' subjective expectations that seroconversion occurred, thus providing a greater incentive for safe sex. We also expected that greater exposure to HIV counseling could change false beliefs about HIV, which could also influence safer sexual behavior. For example, some people believe that they are immune to HIV. If these people are informed that nobody can be immune to HIV, they may act sexually safer in the future. Thus, we measured the impact of repeat HTC on subjective expectations of seroconversion as well as an array of beliefs about important biological and behavioral characteristics associated with HIV. We measured each couple's subjective expectation of whether seroconversion occurred by asking respondents if they believed that the partner who was HIV-negative upon enrollment was now HIV-positive. We expected that couples who received repeat HTC would be less likely to believe that seroconversion occurred.

Next, we had participants report their level of agreement with a series of statements about important biological and behavioral characteristics associated with HIV transmission. Specifically, we asked respondents if they agreed or disagreed with the following questions: 1) Some people can never get HIV even if they have unprotected sex with an HIV positive person; 2) It is impossible for a healthy looking person with no symptoms to have HIV; 3) If an HIV negative person has unprotected sex with an HIV positive person, the HIV negative person will always get HIV; 4) If a person has blood group O, it is impossible for them to get HIV from unprotected sex; 5) If a man is circumcised it is impossible for him to get HIV from unprotected sex; 6) If an HIV positive person is on ARV treatment, it is impossible for them to transmit the virus to an HIV negative person through unprotected sex; 7) A person with a healthy immune system cannot get HIV. We expected couples that received repeated HTC to have more accurate beliefs about HIV and to answer these questions more accurately.

### Statistical Analysis

We conducted a series of statistical analyses to measure the impact of the repeated HTC program. All analyses were at the couple level. We used tests for proportions to measure differences between the treatment and control groups for seroconversion and beliefs (adding controls did not change results, available upon request).

To measure the impact on risky sexual behavior outcomes, we first estimated unadjusted differences in proportions using tests for proportions. Next, we created a panel with two observations for each couple (baseline and after 12 months) and employed a difference-in-differences analysis. This strategy accounts for baseline differences between treatment and control groups and models changes in self reported sexual behavior as function of intervention status. We used a pooled OLS model to assess differences in binary outcomes (“usually” or “ever” having had sex without a condom). To assess differences in categorical outcomes (frequency of sex and condom use) we used ordered probit models, and reported the average marginal effect of receiving repeated HTC for each category.

### Sensitivity Analyses

We conducted a series of sensitivity analyses to test the robustness of our results. First, couples from the treatment group who seroconverted during one of the treatment HTC rounds were not followed up for a post intervention questionnaire on sexual behavior and beliefs. Since these couples seroconverted, it is likely they were practicing particularly risky sex and their exclusion may have caused us to overstate the effects of the program. To account for this, we re-ran analyses with these couples included, with the assumption that they would have reported the riskiest behavior possible for all sexual behavior outcomes, which provided a more conservative estimate.

Next, we tested the sensitivity of our seroconversion results to different assumptions about couples that were lost to follow-up. Since more couples in the treatment group attrited, if attriting couples were more prone to seroconversion, we may have overestimated the value of repeat testing for treatment couples. Similarly, if attriting couples were more prone to safe behavior, we may have underestimated the value of repeat testing. In order to test the range of possible outcomes if there had been no attrition we assumed 4 different scenarios about couples that attrited: 1) All couples in the control group seroconverted and no couples in the treatment groups seroconverted (Upper Bound), 2) no couples that attrited seroconverted (Middle Upper Bound), 3) all couples that attrited seroconverted (Middle Lower Bound), and 4) All couples in the treatment group seroconverted and no couples in the control group seroconverted (Lower Bound).

Next, we tested the sensitivity of our sexual behavior estimates to attrition in a similar way, by assuming that all couples lost to follow-up had the riskiest sexual behavior measures. Since the treatment group was more likely to attrite, this functions as a lower bound.

Next, 32% of the control group couples got retested on their own outside of the intervention, which could cause us to underestimate the result of repeat testing. To account for this we also conduct a two stage least squares instrumental variable analysis, where we instrument the endogenous variable, repeat testing, with our exogenous treatment variable. We would expect the estimated effect of repeat testing to increase under this analysis since it helps to isolate for the causal effect of repeat testing as opposed to the causal effect of being in the treatment group.

Next, since there was a considerable amount of within-couple inconsistencies on self-reported sexual behavior measures, we assessed the robustness of our results to different methodologies for constructing these measures. We tested five different methods of defining sexual behavior measures in place of using the riskiest reported behavior within a couple: 1) safest reported behavior within a couple, 2) male's reported behavior, 3) female's reported behavior, 4) HIV positive partners reported behavior, and 5) HIV negative partners reported behavior (Appendix Table A3).

## Results

Table 1 presents baseline demographic and socio-economic characteristics for the treatment and control group participants that were included in the follow-up questionnaire stratified by males and females. This table demonstrates that our randomization procedure was successful and groups were balanced on important characteristics prior the intervention. There were no statistical differences between groups on any of the characteristics measured.

Table 2 shows differences in sexual behavior measures at baseline between treatment and control groups. We found that there were no statistical differences in sexual behavior measures prior to the intervention, although the treatment group may have been slightly more prone to risky sexual behavior, with a larger portion of couples reporting infrequent condom use.

Only 9 of 246 couples seroconverted after one year, much fewer than expected (Table 3). There was slightly more seroconversion in the treatment group (5.2%) compared to the control group (2.3%), the opposite of what we expected, although the difference of 2.9% was not statistically significant (95% CI -2.0%, 7.9%).

We found that couples that received repeat HTC were less likely to report risky sexual behavior at follow-up. Table 4 shows that the treatment group was 15.6 percentage points less likely to report usually having sex without a condom (p=0.013), 11.9 percentage points less likely to report ever having sex without a condom (p=0.047), and 17.3 percentage points less likely to report never using a condom (p=0.003). There was no difference between groups in frequency of sexual intercourse. Results were similar after adjusting for couple fixed-effects using difference-in-difference models (Table 5). After adjustments, couples in the treatment group were 19 percentage points less likely to report usually having sex without a condom (p=0.010), 14 percentage points less likely to report ever having sex without a condom (p=0.015), 13 percentage points less likely to report never using a condom (p<0.001), and 13 percentage points more likely to report always using a condom (p=0.002). Couples who received repeat HTC also reported less frequent sexual intercourse after adjusting for couple fixed-effects (ordered probit coefficient of 0.545, p=0.026).

We found little difference between treatment and control groups in subjective expectations about seroconversion and false beliefs (Table 6). Contrary to our hypothesis, treatment couples were more likely to report that they believed seroconversion had already occurred, although these results were not significant. There was little indication that repeat HTC had any effect on changing false beliefs, with similar portions in each group agreeing with each false statement.

### Sensitivity Analysis Results

#### Seroconversion

When we include couples that attrited in our seroconversion analysis with different assumptions about the behavior associated with attrition (Appendix Table A4), we found that the upper bound of the effect (all control group and no treatment couples seroconverted) was a nearly 20 percentage point reduction in seroconversion, and the lower bound of the effect (no control group and all treatment couples seroconverted) was a 34 percentage point increase in seroconversion. This wide range between lower and upper bounds further demonstrates that our results for seroconversion are inconclusive.

#### Sexual behavior

We found little change in our sexual behavior estimates when we included treatment group couples that seroconverted during the intervention, with the assumption that they had the riskiest behavior levels (Appendix Table A5). When we defined inconsistencies between partners' sexual behavior measures as the safer behavior reported of the two (instead of the riskiest), we found that the magnitude of our estimates decreased in all cases, and became insignificant for the outcome “usually having sex without a condom” (Appendix Table A6). When we defined inconsistencies between partners as the male's reported sexual behavior, our results were similar to our main model results in Table 5 (Appendix Table A7). When we defined inconsistencies between partners as the female's reported sexual behavior, the magnitude of our estimates decreased in all cases, and became insignificant for the outcome “usually having sex without a condom” (Appendix Table A8). When we defined inconsistencies between partners as the HIV-positive partner's reported sexual behavior, results were similar to our main model results (Appendix Table A9). When we defined inconsistencies between partners as the HIV-negative partner's reported sexual behavior, the magnitude of our estimates decreased in all cases, and became insignificant for the outcome “usually having sex without a condom” and “ever having sex without a condom” (Appendix Table A10).

When we used our treatment variable as an instrument for receiving repeat testing over the year, we found the impact of repeat testing on risky sexual behavior was substantially larger (Appendix, Table A11). The local average treatment affect for usually and ever having sex without a condom was a 37 and 29 percentage point reduction, respectively. The local average treatment affect was also much larger for frequency of condom use with an estimated 40 percentage point reduction in “never” using a condom. These increases in coefficient magnitude add confidence that our results are driven by repeat testing.

Finally, when we assumed that all couples that attrited had the riskiest sexual behavior measures (Appendix, Table A12), we found that our estimates decrease since more treatment group couples attrited. However, the direction of these lower bound estimates remains consistent with our main model, although the magnitude and significance levels decrease. Overall, these sensitivity analyses corroborated our results that repeat HTC may increase condom use, although they added ambiguity to the magnitude of our estimates.

## Discussion

This study was the first to measure the impact of repeat HTC among serodiscordant couples. Overall, our findings are mixed. On the one hand, couples that received repeated HTC generally reported greater condom use, a result that was robust to a variety of estimation methods. However, there was no difference in couples' expectations that seroconversion had occurred at follow-up, which is what we expected to be the pathway to reducing risky behavior. More importantly, there was no evidence that repeat testing reduced seroconversion. Therefore, our findings that repeat HTC reduced risky sexual behavior should be interpreted with caution.

There are a variety of possible explanations for why our results for sexual behavior are not consistent with biological and belief outcomes. First, it is important to note that this study was highly underpowered to detect differences in seroconversion. Previous studies reported much more frequent seroconversion [8,14] and we estimated a 20% annual seroconversion rate from the St. Luke's sample. Given the rarity at which seroconversion occurred in our sample, our estimates are inconclusive.

Second, couples in the treatment group had more interactions with counselors who advocated condom use at each visit. This could have created a greater incentive for treatment couples to report consistent condom use, even if it was not happening in reality. Control couples had less interaction with counselors and may have felt less inclined to appease them with false reports of consistent condom use.

Third, it is possible that the intervention reduced risky sexual behavior within the couple, but increased risky sexual behavior with non-primary partners. This could lead to both increased condom use within the couple and increased HIV incidence in the treatment group.

One aspect that likely dulled the effect of the intervention is that 32% of the HIV negative partners in the control group got an additional HIV test at a health facility sometime between enrollment and follow-up. In other words, many people in the control group sought out the intervention on their own. Although we conducted an instrumental variable analysis to address this issue statistically and get at the causal effect of repeat testing, it is clear that some repeat testing occurs naturally and a costly intervention may not be necessary.

Another possibility for why we do not find evidence that repeat testing prevents seroconversion is that our time period was not long enough. Our theory was that a longer time period between tests increases the perception that seroconversion already occurred. However, it is possible one year is not a long enough duration to see an effect. For example, maybe 4 months compared to 12 months between tests is not a big enough difference, and 4 months compared to 24 months or 36 months would produce a larger effect.

Serodiscordancy among heterosexual couples in Sub-Saharan Africa contributes to a large portion of new HIV transmission in the region. We find evidence that repeat HTC increase (self-reported) condom use, although this effect is inconsistent with our subjective expectation and biological findings. Future work is needed to fully understand whether repeat HTC is an effective tool for reducing seroconversion.

## Figures and Tables

**Table 1:** Balance between treatment and control.

	Males			Females		
	Control	Treatment	p-value	Control	Treatment	p-value
Number of Children	4.3	4.3	0.937	3.3	3.4	0.704
Age	44.7	44.8	0.949	36.1	36.6	0.722
HIV+	44.1%	51.3%	0.257	56.2%	48.7%	0.235
Monthly Income (Kwacha)	12,625	9,181	0.209	3,091	5,414	0.214
**Education**						
No Education	10.9%	7.2%	0.330	16.8%	15.0%	0.702
Primary	62.0%	61.3%	0.905	56.8%	63.6%	0.297
Secondary	27.1%	31.5%	0.455	26.4%	21.5%	0.385
**Work**						
No Work	23.9%	25.9%	0.716	48.1%	49.1%	0.881
Worked Half Time	35.8%	34.8%	0.870	30.4%	29.5%	0.877
Worked Full Time	40.3%	39.3%	0.872	21.5%	21.4%	0.992
**Who Makes Decisions About FP**						
Respondent alone	25.2%	17.9%	0.167	22.1%	21.4%	0.905
Spouse/partner alone	14.8%	10.7%	0.340	14.7%	17.9%	0.503
Respondent and spouse jointly	45.9%	53.6%	0.233	50.0%	44.6%	0.401
Respondent and someone else	0.0%	0.0%	n/a	0.0%	0.9%	0.271
Never discussed	14.1%	17.9%	0.418	12.5%	15.2%	0.542

Statistical differences assessed using tests for proportions. No differences were statistically significant.

**Table 2:** Sexual behavior measures at baseline.

	Control	Treatment	Difference
**Usually Had Sex Without a Condom**	66.9%	70.3%	3.4%
**Ever Had Sex Without a Condom**	83.5%	85.7%	2.3%
**Frequency of Condom Use**			
Never	61.4%	64.2%	2.9%
Sometimes	15.2%	12.8%	-2.3%
Usually	23.5%	22.9%	-0.5%
**Frequency of Sex**			
4+ times per week	23.1%	20.5%	-2.6%
1-3 times per week	50.7%	59.8%	9.1%
couple of times a month	17.9%	14.3%	-3.6%
About once a month	7.5%	5.4%	-2.1%
Less than once a month	0.7%	0.0%	-0.7%

Statistical differences assessed using tests for proportions. No differences were statistically significant.

**Table 3:** Seroconversion.

	Control	Treatment	Difference^1^
Number	3/131	6/115	N/A
Percent	2.3%	5.2%	2.9%
95% Confdence Interval	-0.3% - 4.9%	1.2% - 9.3%	-2.0% - 7.7%

1 Difference estimated using test for proportions

**Table 4:** Sexual behavior after 1 year (unadjusted).

	Control	Treat	Difference^1^ (95% CI)
**Usually Had Sex Without a Condom**	72/135 (53.3%)	43/114 (37.7%)	-15.4%** (-28.0%, -3.2%)
**Ever Had Sex Without a Condom**	99/135 (73.3%)	70/114 (61.4%)	-11.8%** (-23.6%, -.2%)
**Frequency of Condom Use**			
Never	55/135 (40.7%)	27/115 (23.5%)	-17.2%*** (-28.9%, -5.6%)
Sometimes	41/135 (30.4%)	43/115 (37.4%)	7.0% (-4.7%, 18.8%)
Usually	39/135 (28.9%)	45/115 (39.1%)	10.2%* (-1.5%, 23.0%)
**Frequency of Sex**			
4+ times per week	20/135 (14.8%)	10/113 (8.8%)	-6.1% (-14.1%, 2.2%)
1-3 times per week	84/135 (62.2%)	67/113 (59.3%)	-2.9% (-15.1%, 9.3%)
couple of times a month	21/135 (15.6%)	27/113 (23.9%)	8.2%* (-1.5%, 18.2%)
About once a month	7/135 (5.2%)	7/113 (6.2%)	1.0% (-4.8%, 6.8%)
Less than once a month	3/135 (2.2%)	2/113 (1.8%)	-0.5% (-4.0%, 3.1%)

1 Confidence intervals were calculated using tests for proportions

* P<0.1;

** P<0.05;

*** P<0.01

**Table 5:** Panel models with couple fixed effects.

	Marginal Effects	95% Confdence Interval
**Usually Had Sex Without a Condom**^1^	-0.19***	(-0.33, -0.05)
**Ever Had Sex Without a Condom**^1^	-0.14**	(-0.26, -0.03)
**Frequency of Condom Use**^2^		
Never	- 0.137***	(-0.20, -0.074)
Sometimes	0.007	(-0.02, 0.03)
Usually	0.130***	(0.05, 0.21)
**Frequency of Sex**^2^		
4+ times per week	-0.057**	(-0.10, -0.01)
1-3 times per week	-0.034	(-0.07, 0.07)
A couple of times a month	0.047**	(0.004, 0.09)
About once a month	0.036**	(0.00, 0.07)
Less than once a month	0.007	(-0.01,0.01)

1 Difference-in-difference estimates from linear regression with couple fixed-effects

2 Difference-in-difference estimates from ordered probit model with couple fixed-effects

** P<0.05

*** P <0.01

**Table 6:** Subjective expectations and beliefs.

	Male		Female	
	Treat	Control	Treat	Control
**Subjective Expectations**				
Respondent thinks they are HIV positive	17.0%	9.9%	20.7%	11.9%
**Beliefs (% that agree with each statement)**				
Some people can never get HIV even if they have unprotected sex with an HIV positive person	42.2%	44.5%	38.7%	42.2%
It is impossible for a healthy looking person with no symptoms to have HIV	95.5%	96.1%	96.5%	96.3%
If an HIV negative person has unprotected sex with an HIV positive person, the HIV negative person will always get HIV	61.5%	60.6%	71.8%	60.6%*
If a person has blood group O, it is impossible for them to get HIV from unprotected sex	22.6%	26.1%	26.9%	25.2%
If a man is circumcised it is impossible for him to get HIV from unprotected sex	10.1%	6.5%	10.2%	9.8%
If an HIV positive person is on ARV treatment, it is impossible for them to transmit the virus to an HIV negative person through unprotected sex	2.7%	7.9%*	6.3%	8.5%
A person with a healthy immune system cannot get HIV	2.7%	5.2%*	2.7%	5.2%

Statistical differences assessed using tests for proportions

* p<0.1
